# Carney complex presenting with a unilateral adrenocortical nodule: a case report

**DOI:** 10.1186/1752-1947-8-38

**Published:** 2014-02-05

**Authors:** Afsaneh Talaei, Ashraf Aminorroaya, Diana Taheri, Kia N Mahdavi

**Affiliations:** 1 Thyroid Disorders Research Center, Arak University of Medical Science, Arak, Iran; 2 Endocrine and Metabolism Research Center, Isfahan University of Medical Sciences, Isfahan, Iran

**Keywords:** Carney, Cushing’s syndrome, Myxoma, PPNAD, Schwannomas

## Abstract

**Introduction:**

Carney complex is an autosomal dominant syndrome with multiple neoplasms in different sites, including myxomas, endocrine tumors and lentigines lesions. To the best of our knowledge, this is the first report of Carney complex presenting with a unilateral adrenal adenoma associated with a pituitary incidentaloma.

**Case presentation:**

A 27-year-old Iranian woman was referred to our endocrinology clinic with amenorrhea and hirsutism, further confirming a diagnosis of adrenocorticotropic hormone-independent Cushing’s syndrome. The cause was believed to be a right adrenocortical adenoma based on a computed tomography scan. Our patient underwent a right laparoscopic adrenalectomy and pathological examination revealed pigmented micronodular adrenal hyperplasia. Pituitary magnetic resonance imaging also documented a microadenoma that was considered to be an incidentaloma based on normal pituitary function tests. Recurrence of hypercortisolism led to a left laparoscopic adrenalectomy, providing further evidence for the diagnosis of primary pigmented nodular adrenocortical disease. Carney complex was established in light of her history of cardiac myxomas.

**Conclusion:**

We present what we believe to be the first case of Carney complex presenting with a unilateral adrenocortical adenoma in association with a pituitary incidentaloma. Although primary pigmented nodular adrenocortical disease is rare as a component of Carney complex, it should be considered in the differential diagnosis of Cushing's syndrome. Rarely, adrenal and pituitary imaging can be misleading.

## Introduction

Carney complex (CNC) or myxoma syndrome consists of a complex of spotty skin pigmentation (lentigines); myxoma (cardiac, cutaneous), schwannomas and other neoplasms, including testicular tumors and adrenocortical and thyroid follicular carcinomas; and endocrine over-activity, such as primary pigmented nodular adrenocortical disease (PPNAD) [1]. CNC is a rare autosomal dominant disease that affects both sexes equally [2]. About 500 patients have been registered by the National Institutes of Health Mayo Clinic (USA) and the Cochin (France) centers [3]. CNC was first described by Carney *et al*. in 1985 [4].

We report what is, to the best of our knowledge, the first case of CNC presenting with a unilateral adrenal adenoma and pituitary microadenoma, in a 27-year-old woman who was treated with a bilateral laparoscopic adrenalectomy.

## Case presentation

A 27-year old Iranian woman was referred to our endocrinology clinic because of unilateral flank pain. She had a history of hirsutism, acne, amenorrhea and depression. A clinical examination revealed generalized pigmentation, central obesity, moon face, proximal myopathy and hypertension. Our patient’s symptoms and signs were suggestive of Cushing’s syndrome (CS) and she was further evaluated.

Our patient’s medical history included a cerebrovascular event caused by atrial myxomas. She also had a history of two cardiac operations. At the age of 14 years, echocardiography had documented a large left atrial myxoma, and at 19 years old, two large myxomas were identified in her right and left atriums. After her second heart operation, serial echocardiography did not show any myxomas.

During this admission, a hormonal investigation revealed an elevated level of morning plasma cortisol with suppressed plasma adrenocorticotropin hormone (ACTH), elevated urinary free cortisol, loss of circadian rhythm of cortisol secretion, and failure to suppress endogenous plasma cortisol following a low dose dexamethasone suppression test, confirming ACTH-independent CS. Laboratory tests showed that she had a baseline (8 am) plasma cortisol level of 33μg/dL (reference range: 5.1 to 21.9μg/dL), a low dose dexamethasone suppression test result of 26μg/dL, a high dose dexamethasone suppression test result of 21.2μg/dL, an ACTH level of 3 pg/mL (reference range: 10 to 23pg/mL), 24-hour urinary concentrations of vanillylmandelic acid of 2.2mg/day (reference range: <6mg/day); a metanephrine level of 10μg/day (reference range: 30 to 350μg/day); adrenalin level of 5.6μg/day (reference range: 0 to 20μg/day); noradrenalin level of 16.5μg/day (reference range: 15 to 80μg/24h), and a level of insulin-like growth factor 1 of 202ng/mL (reference range: 120 to 485ng/mL). Her other pituitary hormone levels were normal.

**Figure 1:**
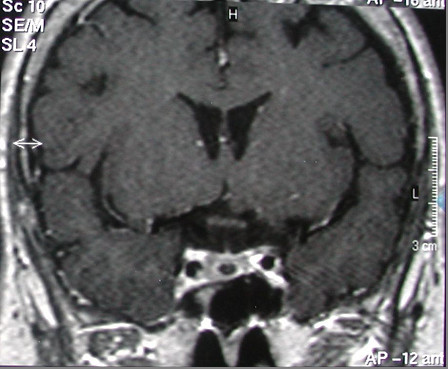
Pituitary microadenoma.

**Figure 2:**
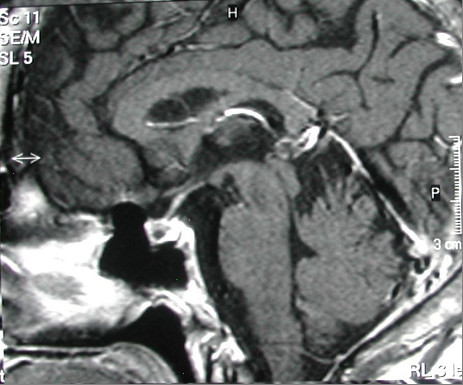
Pituitary microadenoma.

**Figure 3:**
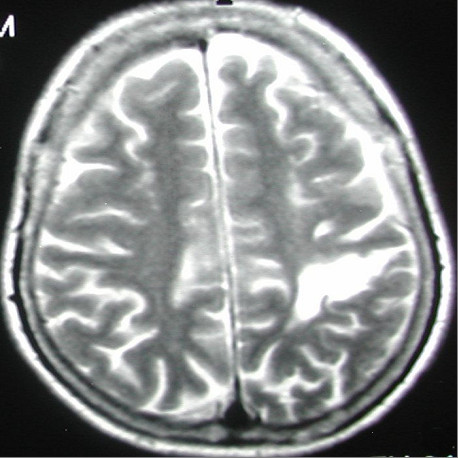
Left parietal lobe infarction.

**Figure 4:**
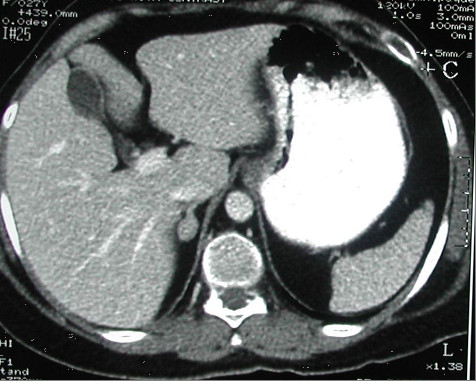
Right adrenal adenoma.

**Figure 5:**
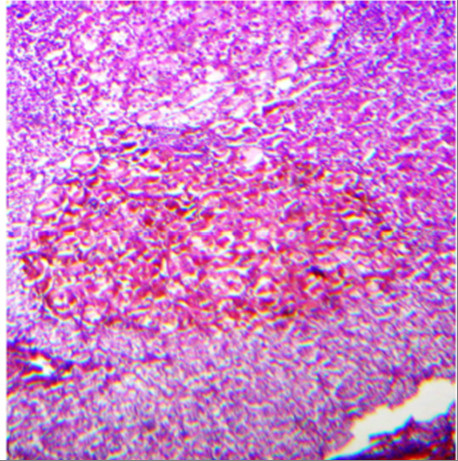
Pathology of the right adrenal adenoma (first operation; large cortical cells with granular eosinophilic cytoplasm containing small nodules of lipofuscin pigments; hematoxylin and eosin stain ×40).

**Figure 6:**
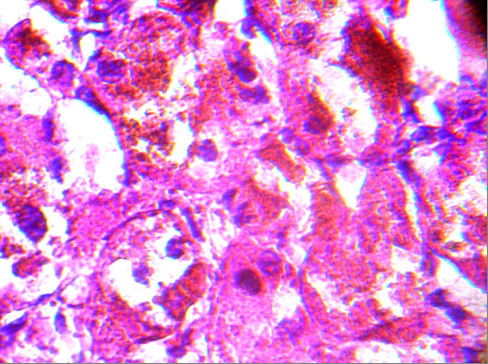
Pathology of the left adrenal adenoma (second operation; cortical cells containing lipofuscin pigments; hematoxylin and eosin stain ×400).

Brain magnetic resonance imaging showed that our patient had a microadenoma (measuring 8×6mm) (Figures 1 and 2) and left parietal infarction (Figure 3). A 15×10mm adenoma of her right adrenal gland was identified in adrenal computed tomography (CT) (Figure 4), whereas her left adrenal gland appeared normal. The hormonal and radiological findings led to a diagnosis of ACTH-independent CS caused by an adrenocortical adenoma of her right adrenal gland. A right laparoscopic adrenalectomy was therefore performed. Pathological examination revealed cortical cell hyperplasia containing small nodules composed of lipofuscin pigment (Figure 5). Postoperative follow-up did not show any improvement in her hypertension or other symptoms. Her plasma cortisol level (12μg/dL) was not suppressed after repeated low dose dexamethasone suppression tests, so a left laparoscopic adrenalectomy was performed and pathological examination revealed the same cortical cell hyperplasia as found in her right adrenal gland (Figure 6). Bilateral adrenal hyperplasia with small pigmented cortical nodules established the diagnosis of PPNAD.

Her plasma cortisol level was suppressed after a dexamethasone suppression test following the second surgery, her hypertension and depressed mood improved and her menses resumed. Because of the PPNAD and her history of atrial myxomas, a diagnosis of CNC was established. An evaluation of other organs, including a thyroid examination and an ovarian ultrasound, were found to be normal. Her family history was negative. None of her family members had any sort of health problems.

## Discussion

We describe the case of a patient with CNC who presented with a unilateral adrenocortical nodule associated with a pituitary incidentaloma, suggesting a diagnosis of ACTH-independent CS. PPNAD was established to be the cause of the CS based on pathological findings. Our patient was then evaluated for CNC. CNC is a multiple endocrine neoplasia consisting of different neoplasms, lentigines, cardiac and cutaneous myxomas, and endocrine over-activity that mainly manifests as hypercortisolism and overproduction of growth hormone.

CNC is an autosomal dominant disorder [5]. It has similarities with McCune-Albright syndrome, multiple endocrine neoplasia and certain kinds of hamartomas, especially Peutz-Jeghers in terms of the mucosal lentigines [6]. CNC is characterized by two or more major manifestations of the syndrome, including lentigines, cutaneous and/or cardiac myxomas, breast myxomatosis, breast ductal adenoma, testicular Sertoli cell tumors, breast tumors, PPNAD, acromegaly, blue nevus, osteochondromyxoma, thyroid carcinoma and mutation of the *PRKAR1a* gene [7]. CNC occurs in a male to female ratio of 43% to 57%. CNC is transmitted through the mother in 43% of cases and through the father in 9% of cases. Most of the patients (70%) of 338 cases were from 67 families, whereas 88 cases had no known affected relative. The genetic origin of the complex could not be definitively determined in 12 cases in Stratakis study. Clinical presentation between family members is variable, which could lead to the absence of a positive family history [2]. Lentigines is the most common presentation of CNC, although it is not consistent [2]. Acromegaly is not common; it is the only pituitary presentation of the disease.

CS caused by PPNAD is more common in endocrine tumors of CNC [8]. PPNAD is a rare cause of ACTH-independent CS. PPNAD may occur in an isolated form or, more commonly, in association with CNC [9]. PPNAD is observed in 25% of patients with CNC, and occurs mostly in children and young people with cases peaking in the second decade. It is very rare in children younger than four years old and is rarely diagnosed after the age of 40 years [2]. Levels of ACTH are low [5]. In one patient out of three, a CT scan of the adrenal glands is normal; in two out of three, micronodules or, more rarely, macronodules in one or both glands can be observed [10]. The nodules are composed of enlarged, globular, cortical cells with granular eosinophilic cytoplasm that contain lipofuscin. Groussin *et al*. described a 2.5cm pigmented macronodule in a patient with isolated PPNAD [11]. In the case of a unilateral macronodule, a non-atrophic contralateral adrenal gland on a CT scan may suggest bilateral disease [12]. Bilateral adrenalectomy is the preferred treatment for CS caused by PPNAD [13].

Myxomas are the most common cardiac tumors in adults and present in 7% of patients with CNC. Cardiac myxomas are thus not a consistent finding of CNC [14] but are responsible for over 50% of deaths in CNC. Early detection for cardiac myxomas using echocardiography is essential, as these tumors can lead to sudden death by embolism, stroke or cardiac failure. Cardiac myxomas are the most common, clinically significant non-cutaneous lesions in patients with CNC [15]. The mean age of patients with myxomas is 26 years old, and 62% of patients are women. The recurrence rate of myxoma is 20% and there is more than one myxoma in 50% of these cases [16].

Almost 50% of patients with CNC are familial cases. In most cases, CNC is caused by inactivating mutations in the gene encoding for the protein kinase A type 1A regulatory subunit (PRKAR1A), which is a tumor suppressor. Mutations in this gene also cause endocrine tumors in CNC [17]. Over 70% of patients with CNC with a classical phenotype show a *PRKAR1A* mutation [18]. *PRKAR1A* mutations were observed in 80% of the familial cases compared to 37% of the sporadic CNC cases. The overall penetrance of CNC in patients with *PRKAR1A* mutations is 97.5% [19]. The routine testing of *PRKAR1A* is not yet recommended [2] and genetic testing was not performed in our patient.

Our patient was considered to have CNC due to the diagnosis of PPNAD and a history of cardiac myxomas. Although this syndrome is inherited as an autosomal dominant trait, our patient had no family history of CNC. Some evidence shows that the disease is genetically heterogeneous. The first patient without a positive family history, similar to our patient, was reported in Japan [20]. The main challenge we faced in correctly diagnosing our patient was that she presented with a right adrenal nodule that was initially supposed to be CS because of an adrenal adenoma; pathological findings and postoperative recurrence of CS confirmed PPNAD. Magnetic resonance imaging also documented a microadenoma that, in association with normal pituitary function tests, was assumed to be an incidentaloma.

A similar case report documented that a 41-year-old man with a clinical presentation of CS and normal adrenal CT had a methyl norcholesterol scan that showed an increased uptake in both adrenal glands. Magnetic resonance imaging also showed a small nodule in his right adrenal gland and a pathological examination of the specimen removed in a right adrenalectomy suggested PPNAD, but hypercortisolism persisted after the operation. A repeat CT showed a 2cm left adrenal adenoma, and his disease improved after a left adrenalectomy. Eleven years later he was diagnosed with papillary cancer of thyroid, which suggests that all patients should be followed for life [21]. Another report outlines the case of an African-American woman with atypical depigmented skin lesions who had undergone seven heart operations because of the recurrence of myxoma [22]. Our patient had undergone two operations because of cardiac myxoma. Guanà *et al*. reported the case of a woman with PPNAD who presented with a unilateral adrenocortical adenoma and improved after both laparoscopic adrenalectomy [23]. Zografos *et al.* in 2010 reported the case of a 27-year-old woman who presented with a left adrenocortical adenoma; a histological examination after a left laparoscopic adrenalectomy revealed PPNAD. Six months later, she underwent right adrenalectomy because of a recurrence of hypercortisolism; histology again revealed PPNAD, although an evaluation for CNC was negative [24].

## Conclusion

As mentioned above, there have been few sporadic case reports of patients with PPNAD who present with a unilateral adrenal adenoma. We present what we believe to be the first case of a patient with CNC presenting with a unilateral adrenal adenoma in association with a pituitary incidentaloma. Although PPNAD is a rare cause of CS, it should be considered in the differential diagnosis of ACTH-independent CS. Rarely, imaging of adrenal and pituitary glands can be misleading. Both laparoscopic adrenalectomy is the preferred treatment in patients with PPNAD.

## Consent

Written informed consent was obtained from the patient for publication of this case report and accompanying images. A copy of the written consent is available for review by the Editor-in-Chief of this journal.

## Abbreviations

ACTH: adrenocorticotropin hormone; CNC: Carney complex; CS: Cushing syndrome; CT: Computed tomography; MEN: Multiple endocrine neoplasia; PPNAD: Primary pigmented nodular adrenocortical disease; PRKAR1A: protein kinase A type 1A regulatory (R1α) subunit.

## Competing interests

The authors declare that they have no competing interests.

## Authors’ contributions

AT was the principal author and major contributor in writing the manuscript. AA collaborated in writing the manuscript. DT performed the histological examination of the adrenal glands. KNM performed the laparoscopic adrenalectomy. All authors reviewed and approved the final manuscript.
